# Neutrophil gelatinase-associated lipocalin does not originate from the kidney during reperfusion in clinical renal transplantation

**DOI:** 10.1186/s40635-021-00422-7

**Published:** 2021-11-22

**Authors:** Arie Passov, Minna Ilmakunnas, Marjut Pihlajoki, Kethe Hermunen, Marko Lempinen, Ilkka Helanterä, Villemikko Kailari, Markku Heikinheimo, Sture Andersson, Eero Pesonen

**Affiliations:** 1 Division of Anaesthesiology, Department of Anaesthesiology, Intensive Care and Pain Medicine, University of Helsinki and Helsinki University Hospital, Topeliuksenkatu 5, PO BOX 266, 00029 HUS Helsinki, Finland; 2 Division of Anaesthesiology, Department of Anaesthesiology, Intensive Care and Pain Medicine, University of Helsinki and Helsinki University Hospital, Haartmaninkatu 4, PO BOX 340, 00029 HUS Helsinki, Finland; 3 Children’s Hospital, Pediatric Research Center, University of Helsinki and Helsinki University Hospital, Stenbäckinkatu 9, PO BOX 347, FIN 00029 HUS Helsinki, Finland; 4 Transplantation and Liver Surgery Clinic, Abdominal Center, University of Helsinki and Helsinki University Hospital, Haartmaninkatu 4, PO BOX 340, 00029 HUS Helsinki, Finland; 5 Faculty of Medicine, University of Helsinki, PO BOX 63, 00014 Helsinki, Finland; 6 Department of Pediatrics, St. Louis Children’s Hospital, Washington University School of Medicine, One Children’s Place, St. Louis, MO 63110 USA

**Keywords:** Neutrophil-gelatinase associated lipocalin, Acute Kidney Injury, Kidney transplantation

## Abstract

**Background:**

Acute Kidney Injury (AKI) is a common clinical complication. Plasma/serum neutrophil gelatinase-associated lipocalin (NGAL) has been proposed as a rapid marker of AKI. However, NGAL is not kidney-specific. It exists in three isoforms (monomeric, homo-dimeric and hetero-dimeric). Only the monomeric isoform is produced by renal tubular cells and plasma NGAL levels are confounded by the release of all NGAL isoforms from neutrophils. Our aim was to investigate whether NGAL is released into blood from injured renal tubules.

**Methods:**

Kidney transplantation (*n* = 28) served as a clinical model of renal ischaemic injury. We used ELISA to measure NGAL concentrations at 2 minutes after kidney graft reperfusion in simultaneously taken samples of renal arterial and renal venous blood. Trans-renal gradients (venous–arterial) of NGAL were calculated. We performed Western blotting to distinguish between renal and non-renal NGAL isoforms. Liver-type fatty acid binding protein (LFABP) and heart-type fatty acid binding protein (HFABP) served as positive controls of proximal and distal tubular damage.

**Results:**

Significant renal release of LFABP [trans-renal gradient 8.4 (1.7–30.0) ng/ml, *p* = 0.005] and HFABP [trans-renal gradient 3.7 (1.1–5.0) ng/ml, *p* = 0.003] at 2 minutes after renal graft reperfusion indicated proximal and distal tubular damage. NGAL concentrations were comparable in renal venous and renal arterial blood. Thus, there was no trans-renal gradient of NGAL. Western blotting revealed that the renal NGAL isoform represented only 6% of the total NGAL in renal venous blood.

**Conclusions:**

Ischaemic proximal and distal tubular damage occurs in kidney transplantation without concomitant NGAL washout from the kidney graft into blood. Plasma/serum NGAL levels are confounded by the release of NGAL from neutrophils. Present results do not support the interpretation that increase in plasma NGAL is caused by release from the renal tubules.

## Background

Acute kidney injury (AKI) occurs frequently in hospitalized patients and is associated with increased mortality [1]. Currently, the diagnosis of AKI requires an increase of serum /plasma creatinine concentrations and/or decrease of urine output over time [2]. For faster diagnosis, neutrophil gelatinase-associated lipocalin (NGAL) has been proposed as early marker of renal injury [3, 4].

Experimental ischaemic kidney injury causes upregulation of NGAL gene expression and protein synthesis, which predominantly seems to occur in renal distal tubular epithelial cells [5–7]. Controversially, in graft biopsies at one hour after reperfusion in human kidney transplantation, NGAL protein accumulation is not found in the distal tubules but instead in the proximal tubular epithelial cells [8]. Since proximal tubular cells seem not to be the primary site of NGAL synthesis [6–8], NGAL detected in proximal tubules must originate from elsewhere. One potential source of NGAL in proximal tubular cells is glomerular filtration and subsequent tubular uptake of circulating NGAL [9]. In experimental studies, proximal tubular cells rapidly take up intravenously administered NGAL and degrade it in lysosomes, and do not release NGAL back into the circulation [7]. Furthermore, some reports suggest that tubular NGAL upregulation does not seem to translate into NGAL release into renal venous blood in experimental ischemic kidney injury [10, 11]. Thus, evidence of NGAL back-leak from renal tubular cells into circulating blood remains elusive [11, 12].

In sepsis, cardiac surgery and exposure to nephrotoxic agents, AKI is associated with high plasma/serum concentrations of NGAL [13]. Likewise, plasma NGAL is elevated in patients with delayed graft function (DGF) after kidney transplantation [14, 15]. These associations are sometimes interpreted as direct evidence that circulating NGAL is derived from renal tubular cells, thus reflecting the severity of tubular injury [7, 14]. However, the presence of NGAL in systemic circulation in AKI and DGF does not imply that NGAL originates from the kidney. First, NGAL is not kidney specific. NGAL is produced also in liver, intestines and lungs [16–18]. Furthermore, neutrophils contain NGAL in their specific granules and release NGAL upon activation [19–21]. Consistent with this, in cardiac surgery, plasma levels of NGAL correlate with neutrophil activation marker lactoferrin [22]. Second, NGAL exists in three isoforms: monomeric (25 kDa), homo-dimeric (45 kDa) and hetero-dimeric (145 kDa) [19, 23]. Mainly the monomeric and to a lesser extent the hetero-dimeric isoforms are produced by the renal tubular epithelial cells [20]. In contrast, neutrophils contain and release all of the isoforms [19–21]. Immunologic methods for NGAL detection, such as enzyme-linked immunosorbent assay (ELISA), that have been used in NGAL research, do not specifically detect the renal isoform [20, 24]. Instead, they detect various combinations of NGAL isoforms based on the configuration of the antibody used in the method [20, 24]. Separating the isoforms by their molecular size with Western blotting revealed that the majority of circulating NGAL in cardiac surgery was of neutrophilic origin [22].

In contrast to the measurement of NGAL from the systemic circulation, kidney transplantation offers a time-controlled and specific clinical model of renal ischaemic injury with the possibility to measure the release of renal tubular injury biomarkers directly from the renal vein. Tubular damage is evident in up to 30% of the cadaveric renal grafts already during procurement, that is even before graft ischaemia [25, 26]. Furthermore, after cold ischaemia but before graft reperfusion, histological acute tubular lesions are present in approximately half of the cadaveric kidney grafts [27]. Graft reperfusion is associated with the washout of renal injury biomarkers and this washout can be detected by obtaining graft venous effluent blood shortly after reperfusion [28–30].

In the present study, we investigated whether NGAL is released from the kidney into circulation after ischaemic injury. Therefore, we measured NGAL concentrations in renal arterial and renal venous blood shortly after graft reperfusion and calculated the NGAL gradient across the renal circulation. In addition, to distinguish between renal and neutrophilic NGAL, the isoforms were separated by their molecular size with Western blotting. Proximal tubular damage biomarker liver-type fatty-acid binding protein (LFABP) and distal tubular damage biomarker heart-type fatty-acid binding protein (HFABP) [31, 32] served as controls for renal tubular damage.

## Methods

### Patients

The study protocol was approved by the Ethics Committee of Helsinki University Hospital (64/13/03/02/2015). Written informed consent was obtained from all patients before enrolment to the study. Twenty-eight adult patients suffering from end-stage renal disease receiving their first renal transplant from a brain-dead donor were prospectively recruited. Exclusion criteria were as follows: previous kidney transplantation, immunosuppression treatment protocol other than the local standard at the time of the study (cyclosporine A, mycophenolate mofetil, methyl prednisolone), significant prior HLA-immunization (panel reactive antibodies over 30%), warfarin therapy, anti-platelet therapy other than aspirin, use of low molecular weight heparins or fondaparinux for other indication than haemodialysis during the last 2 weeks before surgery.

### Donors

All grafts were retrieved from brain-dead heart beating donors. After determination of brain death, all donors received 1000 mg of methyl prednisolone as a bolus. During organ procurement, shortly before organ perfusion, the donors received 30 g of mannitol and 25 000 IU of heparin intravenously. The kidneys were perfused with and preserved in University of Wisconsin solution.

### Anaesthesia and surgery

Anaesthesia and surgery were conducted according to our institutions established clinical practice. Balanced inhalational anaesthesia was used. During reperfusion, mean arterial blood pressure was aimed at 80 mmHg or above. In surgery, the graft venous anastomosis to the recipient’s external iliac vein was completed first. Thereafter, the graft arterial anastomosis to the recipient’s external iliac artery was completed. After reperfusion ureterocystostomy was done. Cold-ischaemia time was defined as the time from the onset of organ perfusion in the donor until declamping of the renal artery in the recipient (graft reperfusion). Warm-ischaemia time was defined as the time from the end of the cold-storage (transfer of the graft from storage-ice to the surgical field) until graft reperfusion.

### Immunosuppression

Immunosuppression consisted of the combination of cyclosporine A, mycophenolate mofetil and methylprednisolone. All patients received peroral cyclosporine A (5 mg/kg) and mycophenolate mofetil (1000 mg) preoperatively. After induction of anaesthesia, 125 mg of methylprednisolone was given intravenously, followed by a second dose shortly before reperfusion.

### Blood samples

Blood samples were drawn at two time points: (T1) after induction of anaesthesia, but before the start of the surgery; (T2) at 2 minutes after reperfusion. At T1, the blood sample was taken from the central venous catheter. At T2, the blood samples were taken across the graft circulation, that is from the arterial blood going into the transplant (the iliac artery) and the venous blood coming out of the transplant (the renal vein). The surgeons took the samples by vessel puncture with a 27G needle. The blood samples were drawn into 5 ml syringes and transferred immediately into pyrogen free citrated vacuum tubes. Plasma was separated by centrifugation for 10 min at 2000G and stored in aliquots at – 80 °C.

### ELISA analyses

Commercial enzyme-linked immunosorbent assay (ELISA) kits produced by Hycult Biotech (Uden, The Netherlands) were used for measurements of NGAL (*n* = 28), HFABP (*n* = 24) and LFABP (*n* = 26). LFABP was used as a positive control of proximal tubular damage and HFABP as a positive control of distal tubular damage [31, 32]. LFABP and HFABP analyses of a few patients are missing due to shortage of either iliac arterial or renal venous plasma samples (Table 3). All DGF patients had LFABP measurements, whereas HFABP measurements were missing from three DGF patients (Table 3).

### Western blot

Non-reducing Western blotting (*n* = 25) was performed on all available samples, to detect plasma NGAL isoforms in preoperative blood sample and in renal venous blood at reperfusion. The method has been described in detail previously [22]. In brief, the samples were mixed 1:4 with Laemmli sample buffer. Protein content was quantified and 10 µg of protein was separated by electrophoresis using Mini-Protean TGX Stain-Free gels (Bio-Rad Laboratories, CA, USA) and transferred onto a polyvinylidene fluoride membrane (Thermo Fisher Scientific, MA, USA). Primary antibody used was a polyclonal rabbit anti-HNL-NGAL (Diagnostics Development, Uppsala, Sweden) at dilution of 1:1000. Normalization and quantification of the protein band intensity were carried out using Image Lab 6.0 software.

### Clinical data

The laboratory and clinical data were collected from the hospital electronic laboratory database and electronic health records. Delayed graft function was defined as a need for dialysis during the first week after transplantation [33]. T-cell mediated acute rejections during the first three months after transplantation were also recorded and defined as deterioration of the graft function associated with typical pathological findings in the graft biopsy.

### Statistical analysis

Data are expressed as number of patients (percent) or median and interquartile range (IQR) when appropriate. Data were analysed with SPSS Version 23 (IBM Corp, Armonk, New York, USA) and GraphPad Prism 7.00 (GraphPad Software, La Jolla, California, USA). For calculation of trans-renal gradients of the biomarkers, the concentration in the arterial sample was subtracted from the concentration in the venous sample. Nonparametric tests were used for continuous data due to the small sample size. Thus, Wilcoxon signed rank test was used for paired comparisons and Mann Whitney *U* test was used for testing differences between the groups where appropriate. Fisher’s exact test was used for comparison of frequencies between the groups. *P-*values less than 0.05 were considered statistically significant.

## Results

### Clinical data

Transplants were retrieved from 25 brain-dead donors (Table 1). The median age of the donors was 60 (53–68) years. Recipient’s characteristics and surgical procedure data are detailed in Table 2. All recipients were undergoing long-term dialysis treatment with either haemodialysis (71%) or peritoneal dialysis (29%), with a median duration on dialysis of 20 (11–38) months. The median graft cold-ischaemia time was 18.7 (15.2–21.7) hours and the median warm-ischaemia time was 54 (48–69) min. Delayed graft function (DGF) occurred in 6 recipients (21%). Four recipients (14%), two of whom had also DGF, suffered from acute T-cell-mediated rejection.

**Table 1:** Donor data

	ALL [N = 25(100%)]
Age (years)	60 (53–68)
Male gender	14 (56%)
Body mass index (kg/m^2^)	24 (22–27)
Hypertension^a^	7 (28%)
Diabetes	1 (4%)
Plasma creatinine at admission to ICU (µmol/l)	53 (47–62)
Cause of brain-death	
Subarachnoid haemorrhage	6 (24%)
Stroke	8 (32%)
Trauma	9 (36%)
Meningitis	2 (8%)
ICU stay (h)^a^	35 (22–81)
Time from declaration of brain death to organ perfusion in the donor (h)	9.4 (7.0–14.0)
Kidney only donors	12 (48%)

Data are median (interquartile range) or number (percentage). ^**a**^Donor data missing from three donors. *ICU* Intensive Care Unit

**Table 2:** Recipient and transplant data

	ALL [N = 28 (100%)]	NO-DGF [N = 22 (79%)]	DGF [N = 6 (21%)]
Age (years)	57 (48–64)	56 (45–62)	62 (54–67)
Male gender	20 (71%)	14 (64%)	6 (100%)
Body mass index (kg/m^2^)	25 (23–28)	25 (23–27)	25 (21–30)
Cause of ESRD			
Polycystic kidney disease	10 (36%)	8 (36%)	2 (33%)
Glomerulonephritis	7 (25%)	4 (18%)	3 (50%)
Diabetes	6 (21%)	6 (27%)	0 (0%)
Other	5 (18%)	4 (18%)	1 (16.7%)
Time on dialysis (months)	20 (10–38)	19 (10–37)	28 (11–65)
Haemodialysis	20 (71%)	14 (64%)	6 (100%)
Peritoneal dialysis	8 (29%)	8 (36%)	0 (0%)
Time on waiting list (months)	11 (4–26)	10 (3–24)	22 (4–37)
Plasma creatinine (µmol/l) at			
1 week after transplant	176 (135–294)	171 (117–223)	580 (265–723)*
1 month after transplant	144 (112–223)	125 (106–175)	211 (188–317)*
3 months after transplant	125 (109–174)	120 (102–172)	155 (143–194)†
Cold ischaemia time (h)	18.7 (15.2–21.7)	19.1 (17.8–21.9)	15.5 (13.0–21.0)
Warm ischaemia time (h)^a^	54 (48–69)	54 (46–69)	60 (49–91)
Rejection at 3 months	4 (14%)	2 (9%)	2 (33%)

Data are median (interquartile range) or number (percentage). ^a^Data missing from three no-DGF transplants; †*p* < 0.05 and *p < 0.01 for DGF vs. NO-DGF. *DGF* Delayed Graft Function. *ESRD* end-stage renal disease

### Trans-renal concentration gradients

**Fig. 1:**
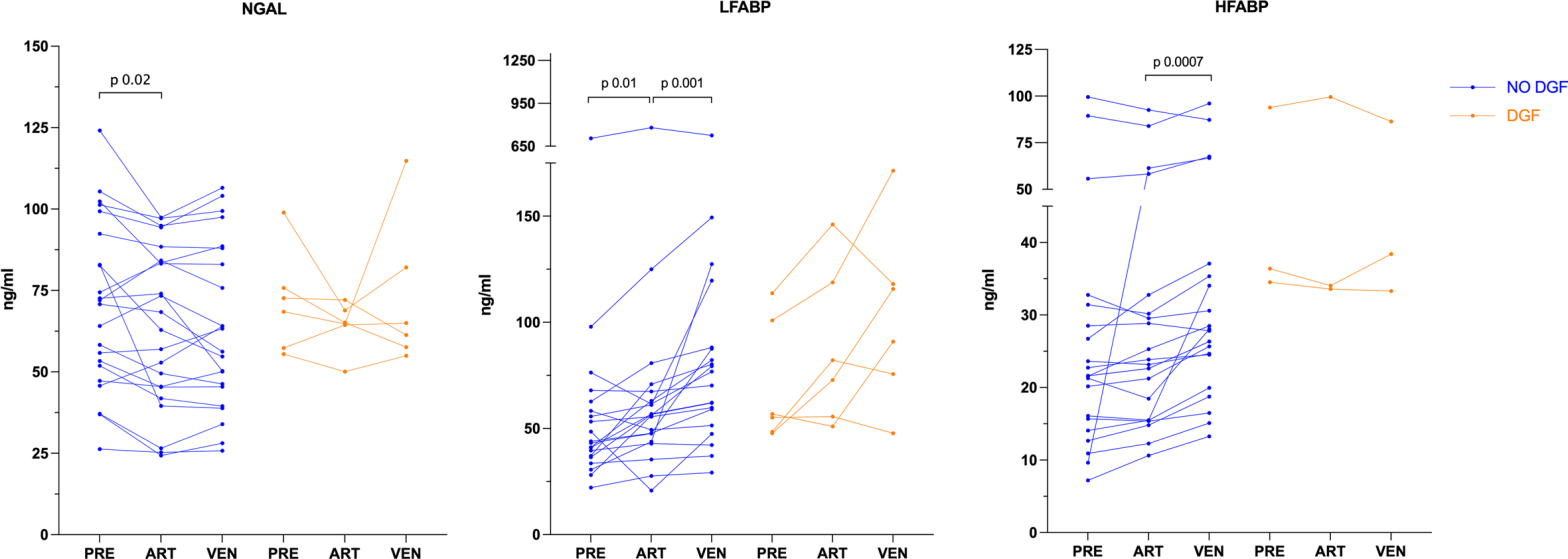
Individual concentrations of NGAL, LFABP and HFABP. *NGAL* neutrophil gelatinase-associated lipocalin, *LFABP* liver-type fatty-acid binding protein, *HFABP* heart-type fatty-acid binding protein, *PRE* pre-operatively, *ART* graft arterial blood, *VEN* renal venous blood; *DGF* delayed graft function; The exact *p*-values of statistically significant results are shown (Wilcoxon signed-rank test). The significance level was 0.05

**Fig. 2:**
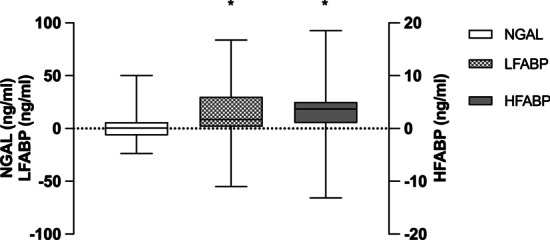
Trans-renal gradients of NGAL, LFABP and HFABP. *NGAL* neutrophil gelatinase-associated lipocalin, *LFABP* liver-type fatty-acid binding protein, *HFABP* heart-type fatty-acid binding protein. *p < 0.01 for renal venous blood vs. renal arterial blood. Trans-renal gradients were calculated as the concentration in arterial blood subtracted from the concentration in renal venous blood. The boxes represent median and interquartile-range and whiskers the lowest and the highest value

The median preoperative LFABP concentration was 48.5 (39.0–64.0) ng/ml (Table 3). At 2 minutes after reperfusion, LFABP levels were significantly higher in renal venous than arterial blood (p = 0.005), indicating outflow of LFABP from the graft (Fig. 1, Table 3). The trans-renal gradient was 8.4 (1.7–30.0) ng/ml (Fig. 2, Table 3).

**Table 3:** Concentrations of NGAL, LFABP and HFABP

	Preoperative venous blood	Renal arterial blood (ART)	Renal venous blood (VEN)	Transrenal gradient (VEN–ART)	*P*-value (ART VS VEN)^§^
**NGAL (ng/ml)**					
ALL (*N* = 28)	71.4 (53.9–90.0)	64.9 (46.5–83.4)	62.3 (47.3–86.7)	0.5 (− 6.4–6.0)	0.585
NO-DGF (*N* = 22)	71.4 (50.7–94.1)	65.6 (44.5–85.3)	59.8 (44.0–88.1)	0.3 (− 4.5–5.3)	0.78
DGF (*N* = 6)	70.6 (56.9–81.6)	64.9 (60.8–69.7)	63.2 (56.9–90.3)	2.7 (− 8.3–22.4)	0.46
*P*-VALUE (DGF VS NO-DGF) ^§§^	0.89	0.98	0.37	0.494	
**LFABP (ng/ml)**					
ALL (*N* = 26)	48.5 (39.0–64.0)	56.7 (47.7–74.8)	78.0 (57.1–116.3)	8.4 (1.7–30.0)*	0.005*
NO-DGF (*N* = 20)	43.5 (36.6–61.6)	56.3 (44.7–66.3)	73.5 (53.3–88.0)	8.4 (2.3–24.1)*	0.001*
DGF (*N* = 6)	56.0 (48.3–104.1)	77.5 (54.5–125.6)	103.3 (68.7–131.4)	16.7 (− 13.0–45.3)	0.345
*P*-VALUE (DGF VS NO-DGF) ^§§^	0.11	0.22	0.083	0.836	
**HFABP (ng/ml)**					
ALL (*N* = 24)	22.2 (15.8–34.1)	24.6 (16.2–33.9)	28.3 (24.6–38.1)	3.7 (1.1–5.0)*	0.003*
NO-DGF (*N* = 21)	21.6 (15.7–28.5)	23.2 (15.5–30.2)	27.8 (24.5–35.3)	3.7 (1.5–5.2)*	0.0007*
DGF (*N* = 3)	36.4 (35.5–65.1)	34.0 (33.8–66.8)	38.4 (35.8–62.4)	− 0.3 (-6.7–2.1)	0.59
*P*-VALUE (DGF VS NO-DGF) ^§§^	0.031*	0.041*	0.122	0.172	

Data are median (interquartile range) for concentrations. All *p*-values are shown; ^§^ART vs. VEN—Wilcoxon signed-rank test; ^§§^DGF vs. no-*DGF* Mann–Whitney *U* test; Transrenal gradient (ART subtracted from VEN). *for statistically significant results (*p* < 0.05); *NGAL* neutrophil gelatinase-associated lipocalin, *LFABP* liver-type fatty-acid binding protein, *HFABP* heart-type fatty-acid binding protein, *ART* concentration in graft arterial blood, *VEN* concentration in graft venous blood, *DGF* Delayed Graft Function

The median preoperative HFABP concentration was 22.2 (15.8–34.1) ng/ml (Table 3). At 2 minutes after reperfusion, HFABP levels were significantly higher in renal venous than arterial blood (p = 0.003), indicating outflow of HFABP from the graft (Fig. 1, Table 3). The trans-renal gradient was 3.7 (1.1–5.0) ng/ml (Fig. 2, Table 3).

The median preoperative NGAL concentration was 71.4 (53.9–90.0) ng/ml (Table 3). At 2 minutes after reperfusion, NGAL levels in renal venous blood were comparable with the NGAL levels in arterial blood, indicating no outflow of NGAL from the graft into venous blood (Fig. 1, Fig. 2, Table 3). The trans-renal gradients of LFABP, HFABP and NGAL did not differ statistically between the DGF and no-DGF patients, although the median gradients of NGAL and LFABP tended to be higher in the patients with DGF (Table 3).

### Western blot analyses

**Fig. 3:**
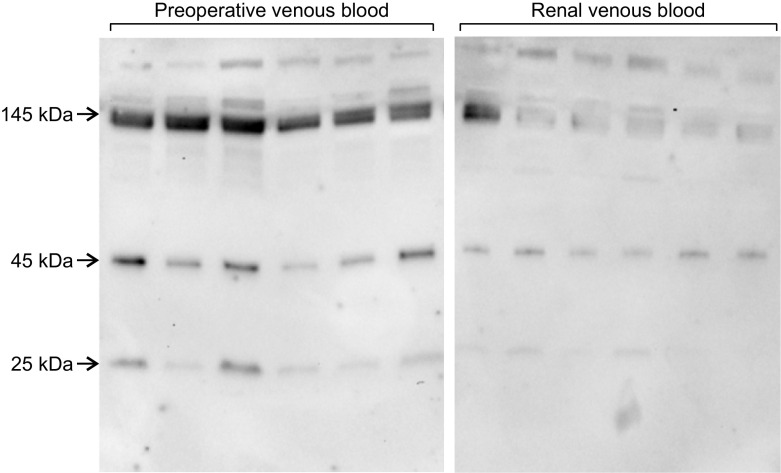
Representative Western-blots of neutrophil gelatinase-associated lipocalin (NGAL) isoforms in six patients. Left panel shows the Western blots of NGAL in preoperative venous blood of six consecutive patients. Right panel shows Western blots of NGAL of the same patients in renal venous blood at 2 minutes after reperfusion of the kidney graft

**Fig. 4:**
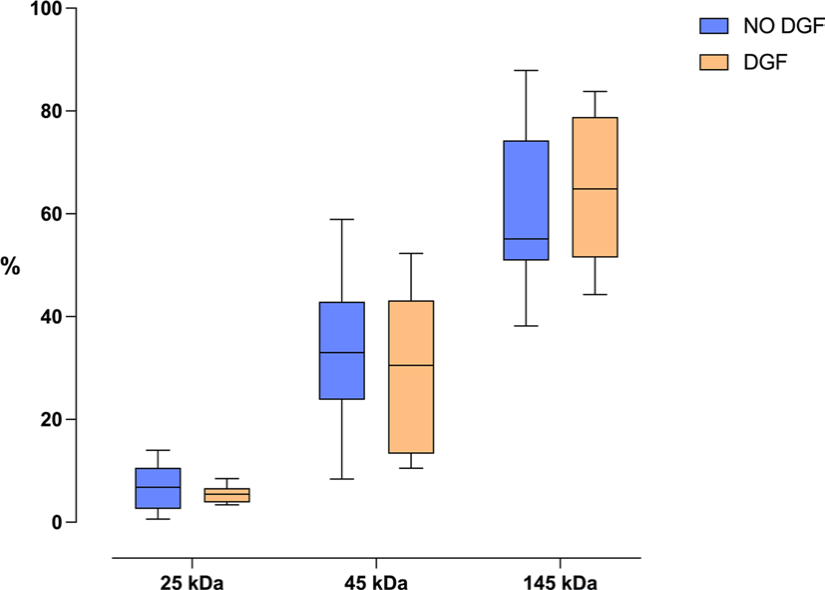
Isoforms of NGAL in renal venous blood in patients with DGF and no-DGF. *NGAL* neutrophil gelatinase-associated lipocalin, *DGF* delayed graft function; The boxes represent median and interquartile-range and whiskers the lowest and the highest value

The Western blot analyses of six representative patients are shown in Fig. 3. The bands of all three NGAL isoforms were clearly visible in all samples. At 2 minutes after reperfusion, homo-dimeric (45-kDa) and hetero-dimeric (145-kDa) isoforms represented the majority of total NGAL in renal venous blood (Table 4). The potentially renal 25-kDa isoform accounted for only 6% of the total NGAL in renal venous blood at 2 minutes after reperfusion (Table 4). The amount of 25-kDa isoform in renal venous blood was lower compared with the amount in preoperative central venous blood (p = 0.007, Table 4). The distribution of NGAL isoforms was comparable in DGF and no-DGF patients (Fig. 4).

**Table 4:** Quantification of NGAL isomers by Western blotting

	Preoperative central venous blood	Renal venous blood
Monomeric, 25 kDa (%)	9.8 (6.4–15.4)	6.1 (3.–9.1)*
Homo-dimeric, 45 kDa (%)	25.4 (15.3–38.7)	32.8 (17.1–40.8)
Hetero-dimeric, 145 kDa (%)	63.4 (48.1–70.2)	58.9 (51.5–76.1)

Data are median (interquartile range). *p < 0.01 for preoperative central venous blood vs. renal venous blood (Wilcoxon signed-rank test). *NGAL *neutrophil gelatinase-associated lipocalin

## Discussion

The origin of circulating NGAL has been under debate [12, 22, 34]. We investigated renal release of NGAL into blood using clinical kidney transplantation as a model of renal ischaemic injury. After reperfusion, there was a significant renal release of proximal and distal tubular injury biomarkers LFABP and HFABP into renal venous blood. In contrast, we found no trans-renal release gradient of NGAL. In addition, Western blot analyses revealed that after reperfusion only 6% of NGAL in the renal venous blood was potentially of renal origin. Also, no increase in the renal venous 25-kDa NGAL occurred after reperfusion. Our results do not support the theory of renal origin of circulating NGAL.

Our study showed significant release of LFABP and HFABP into renal venous blood after reperfusion indicating proximal and distal tubular damage. Both LFABP and HFABP are present in the cytosol of tubular epithelial cells [31], and have been used in several clinical settings as biomarkers of proximal and distal tubular damage, respectively [32]. In contrast, we did not find NGAL release from the kidney grafts into blood as measured by ELISA. Our results are consistent with experimental studies, the results of which have been briefly reported in previous review articles: Despite local NGAL synthesis in tubular cells, no NGAL washout occurred from the kidney into renal venous blood [10, 11].

Interpretation of plasma NGAL concentrations measured by an ELISA assay is complex. Cultured human tubular epithelial cells produce mainly the 25-kDa monomer, while neutrophils produce all of the isoforms [19–21]. To the best of our knowledge, there are no NGAL antibodies which are exclusively specific to the renal isoform [24]. Thus, ELISA assays usually detect various combinations of the isoforms [20, 24]. The ELISA kit used in our study detects all the isoforms. Therefore, it is possible, that the potential washout of the renal isoform could remain undetected with our ELISA assay. To distinguish between different isoforms, we performed Western blot analyses to separate the isoforms by their molecular size [22, 35]. Renal venous sample contained only a median of 6% of the monomeric isoform, i.e. the isoform potentially derived from renal tubules. Furthermore, there was no increase in the proportion of the renal isoform in renal venous sample compared with the preoperative sample. These findings confirm the results of our ELISA assay. All in all, we did not find release of renal tubular NGAL into blood.

Although we did not find renal NGAL release into blood, we do not dispute the association of plasma NGAL levels with AKI and DGF. DGF is a clinical manifestation of acute tubular damage in renal transplantation [36]. Indeed, plasma NGAL concentrations after reperfusion are predictive of DGF [14, 15, 37, 38]. However, the interpretation of renal origin of elevated plasma NGAL in DGF is complicated. In patients undergoing kidney transplantation, NGAL concentrations are elevated preoperatively [14] and often exceed the cutoff value of 165 ng/ml that represents the optimal combination of sensitivity/specificity (Youden index), established for AKI in non-transplantation patients [13]. In non-transplantation patients, NGAL values between the 95% sensitivity threshold and Youden index (71 ng/ml–165 ng/ml) are thought to reflect “grey zone” or increased risk of AKI [4, 13]. By this definition, approximately half of our patients were in the “grey zone” already before transplantation. Only three of these patients ultimately developed DGF. High NGAL levels might be explained by the fact that in patients with end-stage renal disease NGAL, LFABP and HFABP values are chronically elevated and correlate with GFR [39–41]. Therefore, the NGAL cutoff values for kidney injury for non-transplantation patients are not applicable in transplantation setting. For kidney transplantation, higher NGAL cutoff values for DGF at 12 h post-transplantation [14] and on the post-operative day 1 [15] have been published with sensitivity and specificity in a range of 80–90%. Yet, none of the studies have proposed cutoff levels for DGF within the sampling timeframe of early graft reperfusion in the present study. For LFABP the cutoff value in plasma that predicts DGF at post-operative day 1 with 80–90% specificity and sensitivity was 9 mg/ml [42]. All our patients exceeded this value already pre-operatively. To the best of our knowledge, there are no cutoff limits for HFABP in kidney transplantation to predict DGF. Taken together, the levels of plasma NGAL and fatty acid-binding proteins in patients with end-stage renal disease mostly exceed the cutoff values for AKI and are significantly affected by kidney function.

In non-transplantation patients with subclinical kidney injury GFR is compensated via a mechanism called the “renal functional reserve”. Despite compensated GFR, these patients are at increased risk of dialysis, prolonged hospital and ICU-stay and higher mortality [4, 43]. Deceased donors exhibit higher circulating NGAL levels than living related donors [37, 44], and therefore, subclinical kidney injury might be present in the grafts even before procurement. Speculatively, greater release of biomarkers during reperfusion could potentially indicate vulnerable grafts prone to develop DGF. Therefore, we compared the biomarker gradients between patients with DGF and no-DGF. The median gradients of LFABP and NGAL trended to be higher in DGF patients, but the difference was not statistically significant. A larger study is needed to reveal the differences between DGF and no-DGF patients.

The presence of NGAL in the granules of neutrophils poses another important confounding factor in the interpretation of plasma NGAL in patients undergoing kidney transplantation [19, 21]. Activated neutrophils release NGAL and other granule contents in response to inflammatory stimuli [21, 23]. Systemic and local inflammation is inherent to cadaveric renal transplantation [28, 45–47]. Therefore, high plasma NGAL levels in DGF could be a result of neutrophil activation due to systemic and local inflammation instead of indicating damage of the tubular epithelial cells *per se*.

In contrast to plasma NGAL measurement that is affected by GFR [41], urine NGAL might be more reflective of histological tubular damage [48]. Urine NGAL is a good predictor of DGF at 24 h after transplantation with sensitivity of 88% and specificity of 81% [49]. Earlier measurements show clearly less accurate predictive power and low specificity [50]. Our focus was, however, specifically in the earliest events of the graft reperfusion. In this early time-window, measurement of urine NGAL is complicated and prone to several confounding factors. First, a substantial number of patients have ongoing or residual diuresis from the native kidneys. Second, some of the patients remain anuric after graft reperfusion, reflecting potentially severe graft injury and dysfunction. In these patients sampling is impossible. Third, some patients have significant haematuria after surgery, and urine NGAL may thus originate from activated neutrophils. This is an especially important confounding factor of Western blot analyses. Fourth, it may be necessary to flush the bladder catheters to avoid obstruction. Taken together, we decided to refrain from urine analyses, since it is impossible to conclude the origin of NGAL in urine immediately after reperfusion. Furthermore, urine NGAL cannot be measured in anuric patients, who potentially have the worst graft injury and function.

There are strengths and weaknesses in the present study. While the surgeons took the samples by vessel puncture directly from the iliac artery and renal vein, the samples reflect ingoing and outcoming blood in close proximity to the graft. LFABP and HFABP gradients verify that both proximal and distal tubular injury was present at the time of blood sampling. Still, blood samples drawn across the graft reflect concentration differences during only at a given single moment. This sets a limit for detection of renal release of a biomarker. We and others have shown that the washout of biomarkers is best detected during the first few minutes of reperfusion [29, 30, 47]. However, we cannot exclude potential release of NGAL at a later phase due to induced NGAL synthesis in the kidney. Of note, induced protein synthesis indicates cell viability rather than cell injury. Furthermore, experimental data suggest that instead of de novo protein synthesis, NGAL in proximal tubular cells may originate from the blood [7, 9]. In addition, both we and Mishra and co-workers did not investigate the graft biopsies before reperfusion [8]. Thus, it is unclear if NGAL was present in proximal or distal tubular cells at the time of blood sampling. Anyhow, we did not detect NGAL release from the graft despite concomitant proximal and distal tubular injury, as evidenced by renal LFABP and HFABP release. In addition, we acknowledge that our study was not powered to make firm conclusions about the difference between DGF and no-DGF patients or other clinical variables. Finally, we did not analyse urine samples because of potential bias related to sample collection and difficulties in the interpretation of the results in patients undergoing kidney transplantation.

## Conclusions

In conclusion, proximal and distal tubular damage occurs in kidney transplantation without concomitant NGAL washout into renal venous blood after cold and warm ischaemia. Furthermore, circulating NGAL both preoperatively and at early reperfusion is confounded by neutrophils. The NGAL isoform potentially originating from the kidney represents only 6% of circulating NGAL after reperfusion. The proportion of the renal isoform did not increase at the time of reperfusion in the renal venous blood. The present results do not support the interpretation that increase in plasma NGAL is caused by the release from the renal tubules.

## Data Availability

The data sets used and analysed during the current study are available from the corresponding author on reasonable request.

## Abbreviations

AKI: Acute Kidney Injury
DGF: Delayed graft function
ELISA: Enzyme-linked immunosorbent assay
HFABP: Heart-type fatty acid binding protein
IQR: Interquartile range
LFABP: Liver-type fatty acid binding protein
NGAL: Neutrophil gelatinase-associated lipocalin
